# Association study between KIR polymorphisms and rheumatoid arthritis disease: an updated meta-analysis

**DOI:** 10.1186/s12881-019-0754-6

**Published:** 2019-01-29

**Authors:** Hamideh Aghaei, Shayan Mostafaei, Saeed Aslani, Ahmadreza Jamshidi, Mahdi Mahmoudi

**Affiliations:** 1 Rheumatology Research Center, Tehran University of Medical Sciences, PO Box: 1411713137, Tehran, Iran; 2 Department of Immunology, School of Medicine, Tehran University of Medical Sciences, Tehran, Iran; 3 Department of Community Medicine, Faculty of Medicine, Kermanshah University of Medical Sciences, Kermanshah, Iran

**Keywords:** Arthritis, Meta-analysis, Gene polymorphism, killer cell immunoglobulin-like receptor, Disease association

## Abstract

**Background:**

Currently published studies investigating association between the killer cell immunoglobulin-like receptor (KIR) gene polymorphisms and rheumatoid arthritis (RA) reported inconsistent and contradictory results. Hence, we aim to carry out this comprehensive meta-analysis of all eligible studies meeting the inclusion criteria to achieve precise and comprehensive relationships between genetic variations in KIR gene cluster and risk of RA.

**Methods:**

Databases of Medline/PubMed and Scopus were searched to investigate case-control studies prior to May 2018. The associations between KIR gene polymorphisms and RA susceptibility were analyzed by computing the odds ratio (OR) and 95% confidence interval (95% CI) for each study.

**Results:**

A total of 11 comparative case-control studies involving 1847 RA patients and 2409 healthy individuals were included in this meta-analysis. Four significant associations of 2DL3 (OR = 0.591, 95% CI = 0.351–0.994; *P* = 0.047), 2DL5 (OR = 0.716, 95% CI = 0.601–0.853; *P* < 0.001), 2DS5 (OR = 0.623, 95% CI = 0.393–0.988; *P* = 0.045), and 3DL3 (OR = 0.324, 95% CI = 0.129–0.814; *P* = 0.016) genes with decreased RA risk were discovered in this meta-analysis. Although, other KIR receptors including 2DL1, 2DL2, 2DL4, 3DL1, 3DL2, 3DS1, 2DS1-2DS4, and two pseudo gens of 2DP1 and 3DP1 displayed no significant association with predisposition to RA.

**Conclusions:**

These findings provide reliable evidence that 2DL3, 2DL5, 3DL3, and 2DS5 might have a potential protective role for RA.

## Background

Rheumatoid arthritis (RA) is a common chronic autoinflammatory disorder, which is characterized by erosive articular lesions and progressive joint destruction, resulting in irreversible bone deformity and movement disabilities [1, 2]. The overall prevalence of RA ranges from 0.5 to 1% in global populations [3]. It predominantly affects women with a female to male ratio of 2:1. It is believed that the combination of genetic and environmental factors as well as epigenomic contributing factors can break the self-tolerance and provoke immune system against itself during autoimmunity [4–8]. Current studies have reported approximately 60% genetic contribution in susceptibility to RA in various ethnic populations [9–11]. Human leukocyte antigen (HLA)-DR, one of the histocompatibility genes in HLA class II region, is responsible for about 30% of genetic susceptibility to RA [12]. Thus, several genes outside the HLA region are also pivotal for initiation and development of RA. As shown in previous studies, a number of genes, including *PTPN22*, *PADI4*, *STAT4*, *TRAF1*, *TNFAIP3*, *CD40*, and *KIR* have been associated with RA and drawn much attention to investigate more genetic elements contributing to RA risk [9, 13–17].

The essential role of natural killer (NK) cells in RA pathogenesis have been widely investigated in many studies. These cells are present in synovial fluid as well as the blood of RA patients and participate in inflammation and, therefore, damage of joints [18, 19]. In normal physiological conditions, NK cells are able to eliminate target cells with little or no expression of HLA class I molecules on them. Beside cytotoxic function, their ability to secrete cytokines underlines the role of NK cells in development of autoimmune settings [20]. The efficient functional activity of NK cells is governed by three distinct receptor families including, C-type lectin-like group, immunoglobulin (Ig)-like superfamily, and natural cytotoxicity receptors (NCR) [21]. The KIRs (CD 158 family) are expressed mainly on NK cells and a few proportions of T lymphocytes. HLA class I molecules are considered to be the major ligands for KIRs [22]. The cluster of KIR genes is mapped on the leukocyte receptor complex (LRC) locus on chromosome 19q13.4 which is extremely polymorphic among individuals and codes for glycoprotein receptors acting in inhibitory and activating manner [23]. In humans, 17 highly homologous genes have been recognized for KIR genes encoding receptors containing 9 inhibitory, 6 activating, and 2 pseudogenes [24, 25]. KIR receptors are categorized based on the number of their extracellular Ig-like domains (2D or 3D) and whether they have short (S) or long (L) cytoplasmic tail. Two pseudogenes (2DP1 and 3DP1) do not encode functional receptors [26]. Inhibitory KIRs contain long intracellular tails and transduce signals through immunoreceptor tyrosine-based inhibitory motifs (ITIM). On the other hand, activating signals are generated through stimulation of a transmembrane adaptor molecule, namely DNAX activation protein of 12 kDa (DAP12), which possess domains for immunoreceptor tyrosine-based activating motifs (ITAM) [27, 28].

The association of KIR gene polymorphisms has already been demonstrated in several autoimmune disorders [29, 30]. Previously, 11 genetic studies have tried to clarify the role of KIR polymorphisms in susceptibility to RA. Nonetheless, findings from these studies seem to be contradictory in some cases. This discrepancy may be due to small sample sizes, insufficient statistical power and ethnic variations among different populations. Meta-analysis is a statistical method that combines data from multiple researches to establish a reliable estimation. Therefore, in this meta-analysis, we intend to provide a comprehensive evaluation of previously published studies about KIR association with RA susceptibility to have a conclusive and consistent understanding of the role of these molecules in RA pathogenesis.

## Methods

### Literature search strategy

In the current meta-analysis, a systematic literature search was performed to identify relevant published articles in which associations between KIR variations and RA susceptibility were examined (up to May 2018). The electronic databases including Medline/PubMed and Scopus were searched using the following search keywords and subject terms: “The killer cell immunoglobulin-like receptors” or “KIR” or “KIRs”, “rheumatoid arthritis,” or “RA” and “polymorphism” or “variation” or “single nucleotide polymorphism” or “SNP”. All searched studies were retrieved, and their references were also reviewed to discover additional pertinent studies. Original data collected in English language and human population studies.

### Inclusion and exclusion criteria

**Fig. 1:**
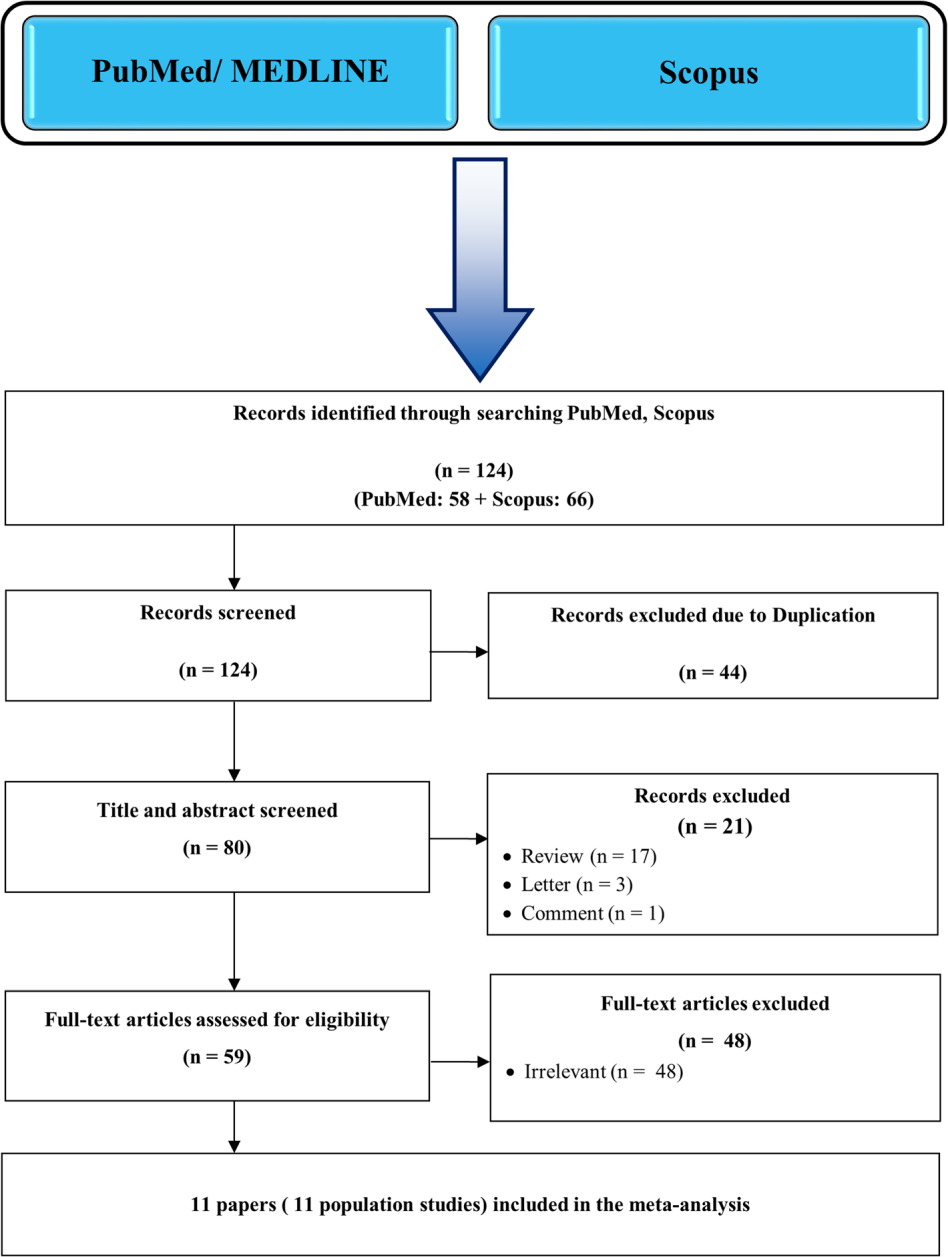
Flow chart of specifications and procedure for the literature search and study selection

A study would be included if met following statements: a) independent case-control studies which assess the association of the KIR polymorphisms and the risk of RA; b) sufficient data for calculating the odds ratios (ORs) and 95% confidence intervals (CIs); c) studies with adequate data representing the genotype and allele frequency of patient and healthy subjects. Accordingly, the exclusion criteria were the following: a) reported duplicate publications and overlapping data b) studies without any information of detailed polymorphism frequencies c) Case reports, letter, review, comment, or abstract that published in journals (Table 1, Fig. 1).

**Table 1:** Characteristics of the included studies in meta-analysis

Author (Ref)	Published Year	Country/Race	Detection Technique	RA Patients	Controls	KIR Polymorphisms
Yen [33]	2001	Western European/Caucasian	PCR	70	76	2DS1, 2DS2
Yen [34]	2006	Taiwanese/Asians	PCR-SSP	122	96	2DL1–3, 2DS1–4, 3DL1–2, 3DS1
Majorczyk [35]	2007	Poland/Caucasian	PCR-SSP	177	243	2DL1–3, 2DS1–5, 2DS4full*001, 2DS4Del*(003,005,006), 3DL1, 3DS1
Middleton [36]	2007	Northern Ireland/Caucasian	PCR-SSOP	331	354	2DL1–5, 2DS1–5, 3DL1–3, 3DS1
Nowak [37]	2010	Poland/Caucasian	PCR	366	690	2DS5
Yasutaka Kimoto [58]	2010	Japanese	PCR-SSP	72	256	2DL1–5, 2DS1–5, 3DL1–3, 3DS1
Ram’ırez-De los Santos [12]	2012	Western Mexico	PCR-SSP	100	100	2DL1–5, 2DS1–5, 3DL1–3, 3DS1, 2DP1, 3DP1
Prakash [38]	2014	North Indian	PCR-SSP	100	100	2DL1–5, 2DS1–5, 3DL1–3, 3DS1, 2DP1
Masoumeh Nazari [39]	2015	Iran/Caucasian	PCR-SSP	400	372	2DL1–5, 2DL5A,B, 2DS1–3, 2DS4–001, 2DS4–003, 2DS5 3DL1–3, 3DS1, 2DP1, 3DP1–001, 3DP1–004
Nishimura [40]	2015	Brazil	PCR-SSP	40	40	2DL3, 2DL5, 3DL1
Velarde-de la Cruz [41]	2016	Western Mexico	PCR-SSP	69	82	2DL1–5, 3DL1–3, 2DS1–5, 3DS1, 2DP1, 3DP1

*PCR* Polymerase Chain Reaction, *PCR-SSP* Polymerase Chain Reaction-Sequence Specific Primer, *PCR-SSOP* Polymerase Chain Reaction-Sequence Specific Oligonucleotide Probes

### Data extraction and quality assessment

The following items were recorded from all 11 eligible studies: first author’s name, publication year, country of origin, ethnicity of the study population, genotyping method, total number of cases and controls, and frequencies of KIR gene polymorphisms. The results were compared and discrepancies were resolved by consensus. For evaluating the quality of the involved studies the Newcastle-Ottawa Scale (NOS) was used [31]. Total NOS scores ranged from 0 to 9. We regarded a study scored 0–3, 4–6, or 7–9 as a low, moderate, or high-quality study, respectively.

### Statistical analysis

The effect size of association between KIR gene polymorphisms and RA susceptibility was determined by odds ratios (ORs) and their 95% confidence intervals (CI) comparing experimental cases to controls for each study. The phenotypic frequency (pf %) of each KIRs was assessed as the percentage of positive numbers between all samples. Genotypic frequency (gf %) among all samples was computed by means of the formula gf = 1– (1 – pf)^1/2^. In the current meta-analysis, heterogeneity among studies was measured by Cochran’s Q-statistic (*P* value< 0.10 was considered statistically significant heterogeneity) and quantified by I-squared (I^2^) tests [31]. *I*^*2*^ values of 25, 50 and 75% were described as low, moderate, and high heterogeneity, respectively. A *P* value> 0.10 or *I*^*2*^ value< 50% demonstrate that the pooled OR was calculated using the fixed-effect model; otherwise, a random-effect model was used. Moreover, we implemented funnel plots to survey the existence of potential publication bias. Egger’s linear regression test and Begg’s test were employed to estimate the funnel plot asymmetry, and *P* < 0.05 was considered to be representative of statistically significant [32]. If heterogeneity presented among studies, sensitivity analysis would be carried out. All statistical analyses were achieved by utilizing STATA (version 14.0; Stata Corporation, College Station, TX) and MedCalc version 13.

## Results

### Characteristics of eligible studies

Regarding to aforementioned inclusion and exclusion criteria, a total of 11 case-control studies reporting the association between KIR gene and RA were available for this meta-analysis. (Shown in Fig. 1) The 11 published studies contained 1847 RA patients and 2409 healthy controls in various ethnic populations including four European, four Asian and three American countries [12, 33–41]. All the recruited papers were published from 2001 to 2016. According to the NOS criteria, all eligible studies had a total score ranging from 7 to 9. A detailed characteristic of the genotype and allele frequencies of various studies involved in this meta-analysis are provided in Table 2.

**Table 2:** Meta-analysis of the pooled association between KIR polymorphisms and RA disease

Polymorphism	No. of studies	RA case n/N	Control n/N	*P-value*	Pooled OR (95% C.I)	Heterogeneity Test (*Q*, *I*^*2*^; *P* - value)	Publication Bias (Begg’s Test, *P*-value; Egger’s test, *P*-value)	Effect Model
2DL1	8	1277/1371	1323/1395	0.736	0.865 (0.372–2.012)	(29.12, 73.8%; *P-value* < 0.001)	(Begg’s Test, 0.53; Egger’s test, 0.51)	Random
2DL2	8	712/1371	748/1439	0.747	1.063 (0.735–1.536)	(35.34, 80.2%; *P-value* < 0.001)	(Begg’s Test, 0.38; Egger’s test, 0.28)	Random
2DL3	9	1224/1411	1295/1435	**0.047**	0.591 (0.351–0.994)	(27.65, 71.1%; *P-value* < 0.001)	(Begg’s Test, 0.99; Egger’s test, 0.24)	Random
2DL4	6	1075/1082	1059/1066	0.864	1.097 (0.382–3.152)	(0.15, 0.0%; *P-value* = 0.99)	(Begg’s Test, 0.09; Egger’s test, 0.001)	Fixed
2DL5	7	614/1112	686/1096	**< 0.001**	0.716 (0.601–0.853)	(8.3, 27.6%; *P-value* = 0.217)	(Begg’s Test, 0.99; Egger’s test, 0.59)	Fixed
2DS1	8	623/1372	651/1389	0.560	0.932 (0.736–1.118)	(14.22, 50.7%; *P-value* = 0.047)	(Begg’s Test, 0.17; Egger’s test, 0.22)	Random
2DS2	9	703/1441	734/1518	0.617	1.068 (0.826–1.380)	(19.98, 59.9%; *P-value* = 0.01)	(Begg’s Test, 0.35; Egger’s test, 0.25)	Random
2DS3	8	372/1371	411/1395	0.176	0.891 (0.735–1.053)	(6.49, 0.0%; *P-value* = 0.483)	(Begg’s Test, 0.90; Egger’s test, 0.60)	Fixed
2DS4	9	1153/1550	1094/1528	0.170	1.150 (0.942–1.404)	(12.57, 36.3%; *P-value* = 0.127)	(Begg’s Test, 0.25; Egger’s test, 0.28)	Fixed
2DS5	8	386/1615	664/1989	**0.045**	0.623 (0.393–0.988)	(52.67, 86.7%; *P-value* < 0.001)	(Begg’s Test, 0.53; Egger’s test, 0.71)	Random
3DL1	9	1296/1411	1338/1435	0.710	0.894 (0.494–1.616)	(25.07, 68.1%; *P-value* < 0.001)	(Begg’s Test, 0.25; Egger’s test, 0.067)	Random
3DL2	7	1182/1204	1149/1160	0.157	0.585 (0.278–1.230)	(1.37, 0.0%; *P-value* = 0.967)	(Begg’s Test, 0.37; Egger’s test, 0.001)	Fixed
3DL3	6	1067/1086	1060/1066	**0.016**	0.324 (0.129–0.814)	(4.28, 0.0%; *P-value* = 0.508)	(Begg’s Test, 0.19; Egger’s test, 0.001)	Fixed
3DS1	8	511/1371	564/1395	0.569	0.917 (0.681–1.235)	(22.14, 68.4%; *P-value* = 0.002)	(Begg’s Test, 0.99; Egger’s test, 0.48)	Random
2DP1	4	633/669	625/654	0.924	0.930 (0.206–4.185)	(14.1, 78.7%; *P-value* = 0.002)	(Begg’s Test, 0.73; Egger’s test, 0.85)	Random
3DP1	3	603/606	575/578	0.976	1.025 (0.206–5.107)	(0.001, 0.0%; *P-value* = 0.99)	(Begg’s Test, NA; Egger’s test, NA)	Fixed

*NA* Not applicable, Values in bold indicate statistically significant differences

### Main results

**Fig. 2:**
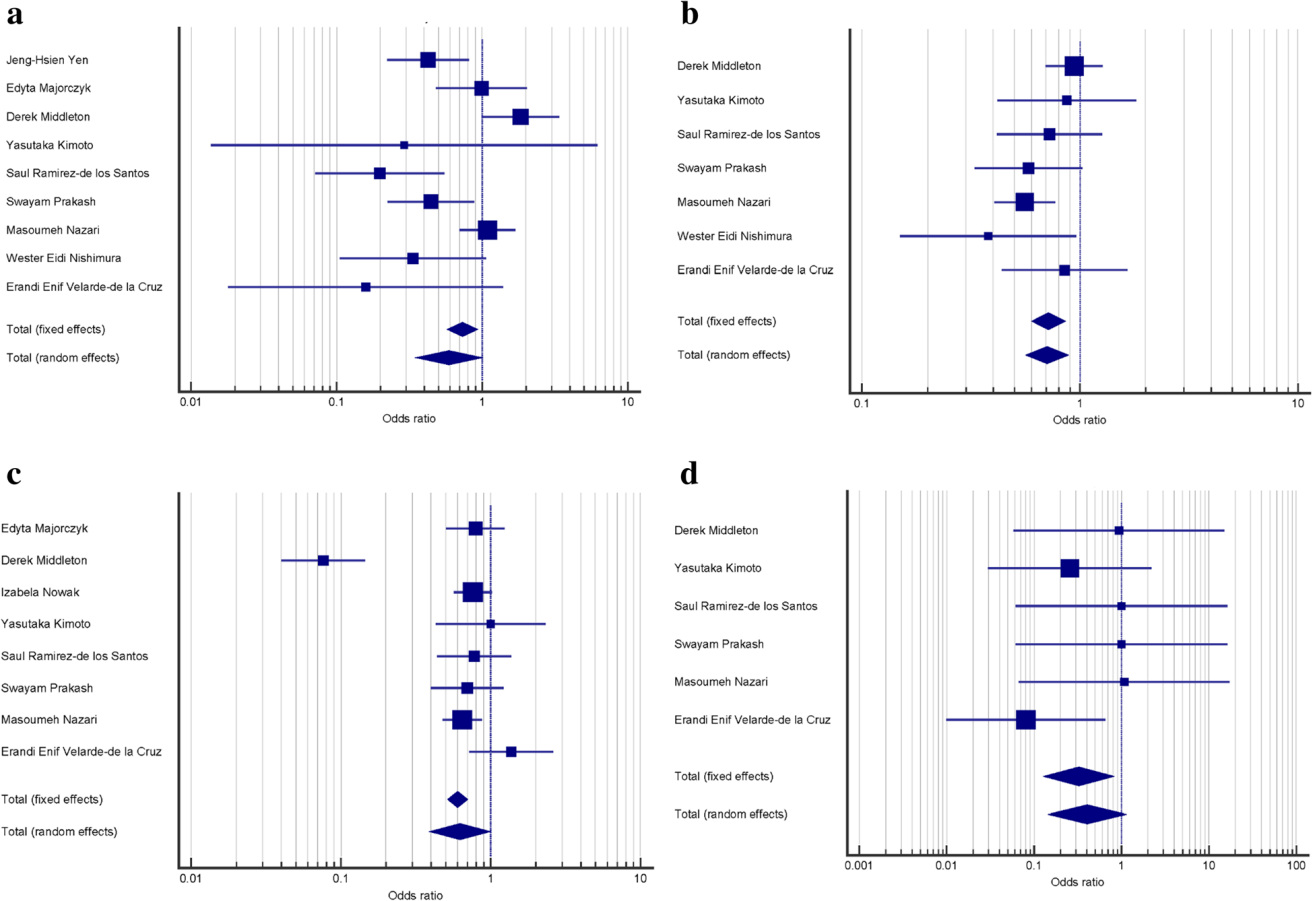
Forest plot. The plot shows results of pooled OR for (**a**) 2DL3, (**b**) 2DL5, (**c**) 2DS5, and (**d**) 3DL3 genes

A summary of meta-analysis findings concerning associations between KIR variations and RA is represented in Table 2. Meta-analysis indicated four markedly significant negative associations of 2DL3 (OR = 0.591, 95% CI = 0.351–0.994; *P* = 0.047), 2DL5 (OR = 0.716, 95% CI = 0.601–0.853; *P* < 0.001), 2DS5 (OR = 0.623, 95% CI = 0.393–0.988; *P* = 0.045), and 3DL3 (OR = 0.324, 95% CI = 0.129–0.814; *P* = 0.016) with RA susceptibility (Fig. 2). Nevertheless, other KIR genes, including 2DL1, 2DL2, 2DL4, 3DL1, 3DL2, 3DS1, 2DS1-2DS4, and two pseudo gens of 2DP1 and 3DP1 showed no significant correlation with predisposition to RA.

### Sensitivity analysis

**Fig. 3:**
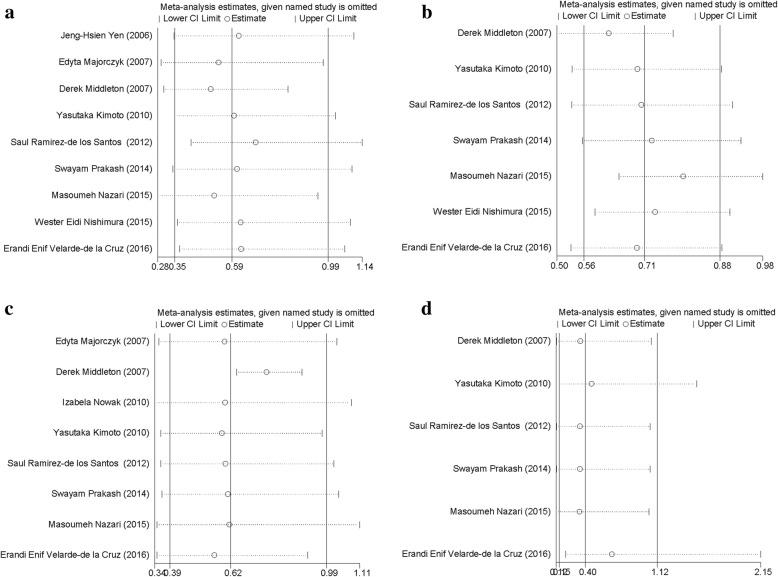
Influence plot. The graph presents sensitivity analysis for (**a**) 2DL3, (**b**) 2DL5, (**c**) 2DS5, and (**d**) 3DL3 genes

A sensitivity analysis was performed by sequential omission of individual and groups of studies. The pooled ORs did not deviate with sequential omission of any participants or group of studies, indicating that our results were statistically robust (Fig. 3).

### Heterogeneity and publication bias

**Fig. 4:**
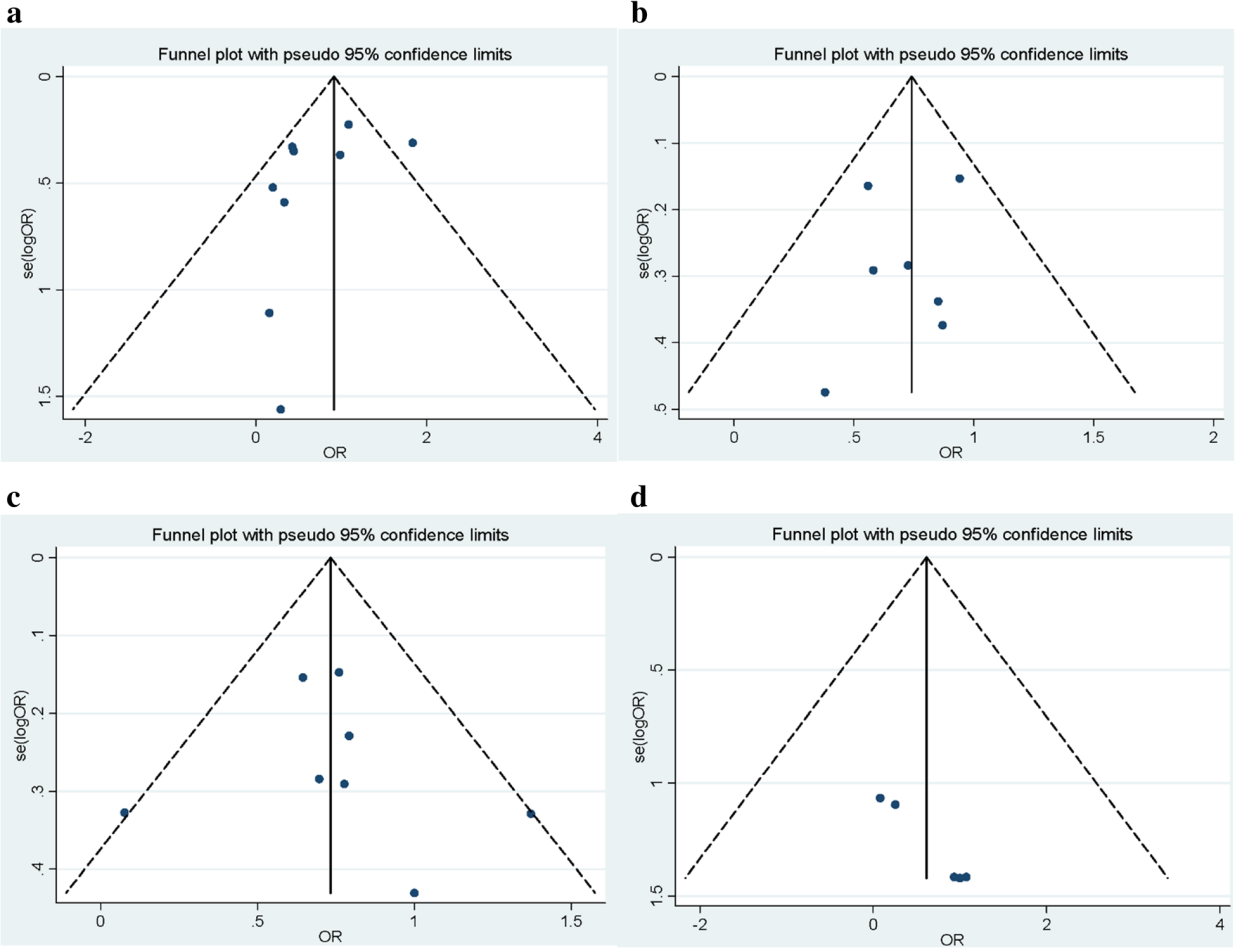
Funnel plot. The plot displays publication bias and heterogeneity of the results between studies for (**a**) 2DL3, (**b**) 2DL5, (**c**) 2DS5, and (**d**) 3DL3 genes

In our meta-analysis, significant inter-study heterogeneity (*I*^*2*^ > 50%; *P* < 0.10) was found in 2DL1 (*I*^*2*^ = 73.8%; *P* < 0.001), 2DL2 (*I*^*2*^ = 80.2%; *P* < 0.001), 2DL3 (*I*^*2*^ = 71.1%; *P* < 0.001), 2DS1 (*I*^*2*^ = 50.7%; *P* = 0.047), 2DS2 (*I*^*2*^ = 59.9%; *P* = 0.01), 2DS5 (*I*^*2*^ = 86.7%; *P* < 0.001), 3DL1 (*I*^*2*^ = 68.1%; *P* < 0.001), 3DS1 (*I*^*2*^ = 68.4%; *P* = 0.002), and 2DP1 (*I*^*2*^ = 78.7%; *P* = 0.002). Therefore, the random-effects model was used to assess the relationship. On the contrary, other KIR polymorphisms revealed no significant heterogeneity and fixed-effects model were utilized to pool the result. Egger’s linear regression test and Begg’s test were applied to check publication bias. Funnel plots also did not show any apparent asymmetry (Fig. 4, Table 2). No publication bias was established for KIR genes in overall analysis (Egger’s regression test *P* > 0.1).

## Discussion

We intend to perform this meta-analysis to achieve a comprehensive analysis of the previously published studies about KIR association with RA susceptibility to figure out a conclusive and consistent understanding of the RA pathogenesis underlying KIR gene polymorphisms. The analysis revealed significant association of 2DL3, 2DL5, 2DS5, and 3DL3 genes, which decreased RA risk.

RA is a common autoimmune disorder, which mainly affects the joints and brings about bone erosions. One subject of debate at present is the role of genetic elements, which are involved in the initiation and development of RA. Integration analysis of genome wide association study (GWAS) successfully reported newly natural killer cell mediated cytotoxicity pathway as a contributing genes which are associated with RA [42].

Human NK cells are CD3^−^CD56^+^ lymphocytes of innate immune system and are subdivided into CD56^bright^ and CD56^dim^ phenotypes based on CD56 expression level. While CD56^dim^ NK cells participate in cytotoxic responses, CD56^bright^ subsets are highly involved in cytokine secretion especially interferon (IFN)-γ. According to previous researches, following diminished frequency of NK cells in peripheral blood, CD56^bright^ subsets could migrate through blood vessels to inflamed joints and propagated in the synovial fluid of RA patients [43, 44]. Enhancement of NK cells in synovial fluids resulted in high amount of IFN-γ production which, in turn, contributed to perpetuation of inflammatory milieu in this autoimmune disorder [45]. Of note, IFN-γ per se has ability to activate surrounding macrophages through cell-cell contact to release more inflammatory mediators, mostly tumor necrosis factor (TNF)-α which reinforce inflammatory environment in arthritic joints [46].

NK cells are also responsible for killing target cells that are not able to express self-MHC molecules [47]. Their function is highly modulated by several activating and inhibitory membrane receptors, particularly KIRs [26]. In 2001, the first case-control research regarding association of KIRs and RA undertaken by Yen et al. that showed the KIR2DS2 involvement in the development of rheumatoid vasculitis [33]. From 2001 up to now, several studies were carried out to delineate the relationship of KIR genes repertoire with susceptibility to RA in diverse ethnic populations, though the findings appear inconsistent and conflicting. Thus, we decided to fulfill this comprehensive meta-analysis by incorporating data of 11 relevant studies in order to reevaluate the interest associations. The overall results divulged that KIR2DL3, KIR2DL5, KIR3DL3, and KIR2DS5 were negatively associated with susceptibility to RA, providing evidence that KIRs might potentially affect RA progression. On the other hand, other KIR genes, including 2DL1, 2DL2, 2DL4, 3DL1, 3DL2, 3DS1, 2DS1-2DS4, and two pseudo gens of 2DP1 and 3DP1 displayed no significant association with RA risk in our study.

KIR genes consist of two haplotypes; The “A” haplotype contains inhibitory genes and one activating gene (KIR2DS4) with protective effect against autoimmunities. “B” haplotype, on the other hand, exhibits more activating gens (KIR2DS1, -2DS2, -2DS3, -2DS4, -2DS5, and -3DS1) and inhibitory genes, KIR2DL2 and KIR2DL5 that increase the risk of autoimmune disorders [24, 26, 48]. It is widely accepted that the proper activation of NK cells depends on the equilibrium of activating and inhibitory KIR receptors. Considering their high degree of polymorphisms in two levels of gene content and allelic variations, they are mostly related to many autoimmune diseases, such as RA, ankylosing spondylitis, psoriasis vulgaris, scleroderma, SLE, diabetes mellitus, Crohn’s disease, Behçet’s disease, and etc. [21, 39, 49–54].

In this study, KIR2DL3 showed negative association with the RA risk and in agreement with the results of Parkash et al. and Ramirez-De los Santos et al. studies, it provided protection against RA [12, 38]. In this context, it has been clarified earlier that KIR2DL3 inhibits the production of cytokines like IFN-γ. Of note, NK cells-derived IFN-γ is a fundamental source for Th1 priming in RA [55]. Therefore, lack of IFN-γ secretion by NK cells could reduce T cells autoreactivity [56].

In addition, our results also suggest that KIR2DL5 and KIR2DS5 were protective against RA. KIR2DL5 and KIR2DS5 both are present at haplotype B, but they function differently in the inhibitory and activating manner, respectively. Meanwhile, their respective ligands have not been discovered successfully [57].

Interestingly, KIR2DS5 display protective effect in some diseases, despite an activating function. A study by Nowak et al. showed that KIR2DS5 plays a protective role in ankylosing spondylitis, endometriosis, and acute kidney graft rejection with no significant effect in RA. According to their literature, this paradoxical protective effect is likely related to the pronounced role of KIR2DS5 in tolerance induction and, therefore, prevention of autoimmune conditions [37]. It should be noted that this phenomenon might be due to linkage disequilibrium (LD) with some neighboring inhibitory loci in KIR haplotype. Thus, LD can contribute to modulate gene expression and give explanation as a putative cause of protective effect of KIR2DS5.

It has been reported previously that KIR2DL5 significantly had protective effect in SLE patients. Due to inhibitory signals from KIR2DL5, NK cells functions can be suppressed in the onset of SLE. Moreover, the less prevalence of KIR2DL5 associated with the increased predisposition to overall infections in SLE patients [58]. Although their findings could not support the relation of KIR2DL5 in RA individuals, we observed a highly significant association of KIR2DL5 with risk of RA in our population study, which may impress the disease susceptibility in the same manner as does in SLE.

KIR3DL3 is one of the so-called “framework” genes, which is present in all KIR haplotypes. It is located in the centromeric half of haplotype at 5’-end and its ligand is uncertain yet [59]. This inhibitory receptor can prohibit NK cell cytotoxicity via single ITIM motif in its cytoplasmic tail. Unlike previous reports, we revealed that a negative association exist between KIR3DL3 and RA risk.

A meta-analysis undertaken by Li et al. in 2015 to evaluate association between KIR variations and RA risk in East Asians and Caucasians subpopulations. The findings of their survey represented two positive associations, including 2DL1, 2DS1, and 2DL3 as a protective factor for RA risk only in East Asians, but not in Caucasians [60]. In the current study, we incorporated more studies with further ethnic populations to achieve better results. The only consistence results obtained from current meta-analysis and that of Li et al. study, was 2DL3 as protective factor in RA patients [60]. While the previous meta-analysis found two positive associations of KIR genes with RA susceptibility, we found that all significantly associated KIR genes had negative association with RA risk. KIR2DL5, KIR2DS5, and KIR3DL3 conferred a protective role in RA development [60].

This meta-analysis provides comprehensive information from 11 case-control studies to better understanding of KIR and RA association, but some shortcomings should be considered. First, between-study heterogeneity was observed which might distort analysis. Second, the number of published studies entered in this research was relatively small which restrict the estimation power of the present study. However, considering the increased number of subjects included in this meta-analysis, the obtained results are more reliable than the previous analysis [60]. Additionally, various populations of Europe, Asia, and American nations were included in the current meta-analysis, increasing the general conclusion of the results to populations worldwide.

## Conclusion

In consideration of all, present meta-analysis identified that‌ KIR2DL3, KIR2DL5, KIR2DS5, and KIR3DL3 seem to have a potential protective role in RA risk. However, premised on forenamed limitations, further research studies are still required to explore the association between the KIR polymorphisms and RA risk with a larger sample sizes and controls in order to validate these findings.

## Abbreviations

CI: Confidence interval
Gf: Genotypic frequency
GWAS: Genome wide association study
HLA: Human leukocyte antigen
IFN: Interferon
Ig: Immunoglobulin
ITAM: Immunoreceptor tyrosine-based activating motifs
ITIM: Immunoreceptor tyrosine-based inhibitory motifs
KIR: Killer cell immunoglobulin-like receptor
LD: Linkage disequilibrium
LRC: Leukocyte receptor complex
NCR: Natural cytotoxicity receptors
NK cells: Natural killer cells
NOS: Newcastle-Ottawa Scale
OR: Odds ratio
RA: Rheumatoid arthritis
TNF: Tumor necrosis factor
